# Conformational dynamics of dynamin-like MxA revealed by single-molecule FRET

**DOI:** 10.1038/ncomms15744

**Published:** 2017-05-26

**Authors:** Yang Chen, Lei Zhang, Laura Graf, Bing Yu, Yue Liu, Georg Kochs, Yongfang Zhao, Song Gao

**Affiliations:** 1 State Key Laboratory of Oncology in South China, Collaborative Innovation Center for Cancer Medicine, Sun Yat-sen University Cancer Center, Guangzhou 510060, China; 2 National Laboratory of Biomacromolecules, CAS Center for Excellence in Biomacromolecules, Institute of Biophysics, Chinese Academy of Sciences, Beijing 100101, China; 3 Institute of Virology, Medical Center, Faculty of Medicine, University of Freiburg, Freiburg 79008, Germany; 4 Spemann Graduate School of Biology and Medicine, University of Freiburg, Freiburg 79104, Germany

## Abstract

Human myxovirus resistance protein 1 (MxA) restricts a wide range of viruses and is closely related to the membrane-remodelling GTPase dynamin. The functions of MxA rely on domain rearrangements coupled with GTP hydrolysis cycles. To gain insight into this process, we studied real-time domain dynamics of MxA by single-molecule fluorescence resonance energy transfer. We find that the GTPase domain-bundle-signalling-element (BSE) region can adopt either an ‘open' or a ‘closed' conformation in all nucleotide-loading conditions. Whereas the open conformation is preferred in nucleotide-free, GDP·AlF_4_^−^-bound and GDP-bound forms, loading of GTP activates the relative movement between the two domains and alters the conformational preference to the ‘closed' state. Moreover, frequent relative movement was observed between BSE and stalk via hinge 1. On the basis of these results, we suggest how MxA molecules within a helical polymer collectively generate a stable torque through random GTP hydrolysis cycles. Our study provides mechanistic insights into fundamental cellular events such as viral resistance and endocytosis.

## Abstract (web summary)

MxA (myxovirus resistance protein A) is a viral restriction factor whose activity depends on self-assembly into polymeric rings and helical filaments. Here the authors reveal the conformational movements involved in generating torque within polymeric MxA molecules and the dynamic conformational changes that occur upon GTP loading and hydrolysis.

Myxovirus resistance (Mx) proteins are key mediators of interferon-induced innate immune response in vertebrates^1^,^2^,^3^. Humans have two Mx proteins, MxA and MxB, which share 87% sequence similarity and are engaged in viral restriction^1^,^4^,^5^. MxA restricts a broad range of RNA viruses^6^,^7^,^8^, including the influenza virus^5^,^9^, whereas MxB was recently shown to inhibit human immune deficiency viruses^10^,^11^,^12^. It has been suggested that MxA recognizes the nucleoproteins of target viruses and polymerize around the viral ribonucleoparticles^13^,^14^, thereby disrupting viral replication^15^,^16^,^17^. In addition, MxA was also reportedly associated with the endoplasmic reticulum-Golgi intermediate compartment^18^,^19^. However, the detailed antiviral process of MxA and MxB still remains elusive.

MxA is closely related to dynamin which catalyses membrane fission in various essential cellular events including endocytosis^20^,^21^,^22^. Both dynamin and MxA are able to tubulate liposomes *in vitro*^18^,^23^,^24^. They have similar architectures featured by the N-terminal GTPase domain (GD), a bundle-signalling-element (BSE) composed of three helices from widely dispersed sequence regions, and a four-helical stalk that is connected to the BSE via two loops named hinge 1 (refs 17, 25, 26, 27). Their functions depend on self-assembly into polymeric rings or helical filaments via stalk, and association between the GDs from neighbouring rings/rungs^17^,^23^,^25^,^26^,^27^,^28^. Structural studies on stalkless-MxA and the GD-BSE construct of dynamin revealed that GTP binding and hydrolysis are coupled with relative movement between GD and BSE^28^,^29^,^30^. Whether this dramatic conformational change occurs in full-length MxA is unknown.

It has been suggested that a hydrolysis-dependent powerstroke mediated by BSE drives the constriction of the dynamin helical filament^30^. Final membrane fission is preceded by a hemi-fission state^31^ and requires the disassembly of dynamin polymer dependent on GTP hydrolysis, in which the rearrangement of pleckstrin homology (PH) domain against the stalk is thought to be involved^31^,^32^. On the other hand, the relative movement between BSE and stalk has not been investigated and whether this also plays a role in regulating disassembly of the polymer is not understood.

Here we combined X-ray crystallography with fluorescence resonance energy transfer (FRET)^33^,^34^,^35^,^36^,^37^ to reveal the conformational dynamics of MxA in different nucleotide states at the single-molecule level. Our data indicate that MxA molecules display specific conformational preferences and potentials of domain rearrangement in the GD-BSE and BSE-stalk regions that are regulated by GTP binding and hydrolysis. In addition, we show that the intact hinge 1-BSE interaction is an essential pivot that underlies the flexibility of MxA. Our results provide insight into the mechanisms of MxA and related dynamin-like proteins by explaining the behaviours of component molecules within the MxA polymer during GTP turnover.

## Results

### Crystal structure of a monomeric MxA

**Figure 1:**
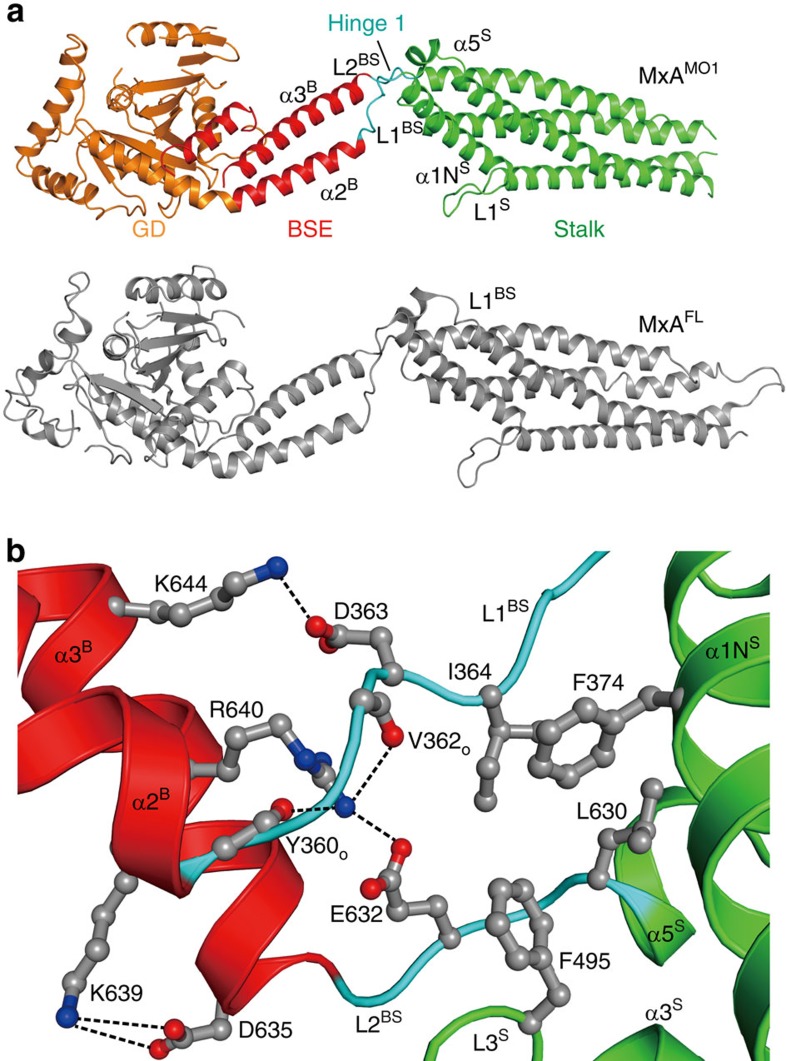
Crystal structure of MxA^MO1^. (**a**) Structural comparison between MxA^MO1^ (upper) and MxA^FL^ (lower, PDB accession number 3SZR). For MxA^MO1^, GD, BSE, stalk and hinge 1 are coloured orange, red, green and cyan, respectively. Secondary structure elements surrounding hinge 1 are indicated. MxA^FL^ is coloured grey. (**b**) Associations of hinge 1 with the BSE and stalk. Involved residues are shown in ball-and-stick representation. Domains are coloured as in **a**.

MxA forms stalk-mediated tetramers in solution, which can further polymerize with increased protein concentration or the addition of GTP^25^. To allow the use of single-molecule FRET (smFRET) for the study of the intramolecular dynamics of MxA, we generated several constructs (Supplementary Fig. 1) based on a monomeric mutant M527D. This mutant retains the ability to form dimers via the GDs in the presence of the transition state mimic GDP·AlF_4_^−^ (ref. 38). To verify their architectures, we determined a 2.9 Å nucleotide-free crystal structure of a monomeric MxA construct (termed MxA^MO1^) containing the following modifications: (i) deletion of N-terminal 32 residues and part of L4^S^ (residues 533–561 inclusive), (ii) point mutations in interface 1 (I376S, K614S, L617S and L620S) and interface 2 (M527D) (Fig. 1a, Supplementary Figs 1 and 2a and Supplementary Table 1).

As expected, inside the crystal, the MxA^MO1^ does not form criss-cross linear oligomers as in the stalk^25^ and near-full-length MxA^FL^ (ref. 17) structures of MxA, suggesting that the oligomerization interfaces were successfully disrupted (Supplementary Fig. 2b). The overall folding and domain distribution of MxA^MO1^ shows a high consistency with MxA^FL^ (Fig. 1a). Moreover, the infrastructure of MxA^MO1^ hinge 1 remains intact, and a higher quality of diffraction data compared to that of MxA^FL^ revealed that the previously unexplained K644 of α3^B^, a conserved residue among Mx proteins, is involved in the BSE-hinge 1 association by forming a salt bridge with D363 of L1^BS^ (Fig. 1b, Supplementary Figs 2c and 3). Altogether, the MxA^MO1^ structure indicates that disruption of the oligomerization interfaces of the stalk does not affect the architecture of MxA.

### Strategy for smFRET experiments

**Figure 2:**
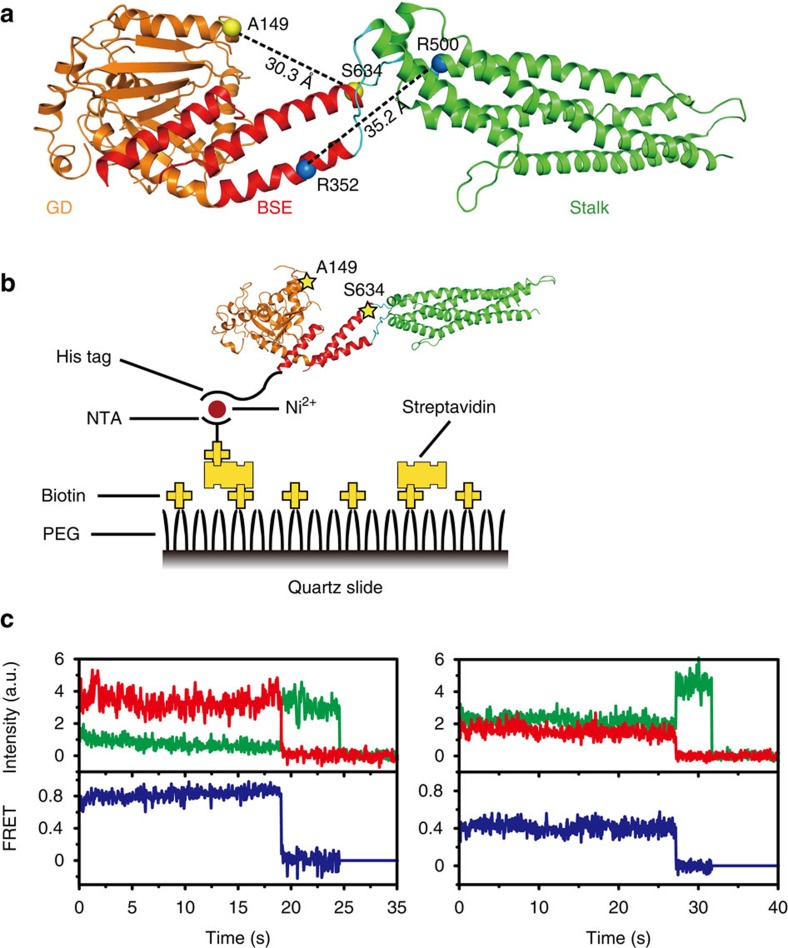
Strategy of the smFRET assay. (**a**) Selected cysteine (Cys) pairs for dye labelling. Positions of A149C/S634C and R352C/R500C pairs are specified with yellow and blue spheres, respectively. Distances between the C_β_ atoms in each Cys pair are indicated. Domains of MxA^MO2^ are coloured as in Fig. 1a. (**b**) Immobilization strategy. His_6_-tagged MxA^MO2^-AS (shown here as an example for all measured samples) labelled with Cy3 and Cy5 (stars) was immobilized via biotin-NTA-Ni^2+^ on a PEG/biotin-PEG-coated quartz slide treated with streptavidin. (**c**) Representative traces (Cy3 as donor in green, Cy5 as acceptor in red, and FRET in blue) from the assay on MxA^MO2^-AS in the nucleotide-free condition.

To investigate the domain movement of MxA by smFRET, we individually mutated A149/S634 pair and R352/R500 pair to cysteines for site-specific labelling with Cy3/Cy5 (ref. 39) on a monomeric MxA construct (MxA^MO2^) whose solvent-exposed inherent cysteines were replaced by other amino acid residues (see Methods, Fig. 2a and Supplementary Fig. 1). These mutants, termed MxA^MO2^-AS (MxA^MO2^-A149C/S634C) and MxA^MO2−^RR (MxA^MO2^-R352C/R500C), retained near-normal properties of guanine nucleotide binding, GTPase activity and G-domain-mediated dimerization in the transition state with respect to the M527D mutant^25^,^38^ (Supplementary Fig. 4a–c). In minireplicon assays, the A149C/S634C double mutant showed wild-type antiviral activity, whereas the R352C/R500C double mutant did not (Supplementary Fig. 5). Nevertheless, according to the results of the above biochemical analysis, the protein folding should not be perturbed by these mutations. Moreover, all selected sites were labelled with high efficiency (>80%), low nonspecificity (<5%) and minor fluorescent anisotropy, indicating that these mutants were appropriate for smFRET studies (Supplementary Fig. 6a,b). For smFRET experiments, N-terminally His_6_-tagged MxA with Cy3/Cy5- labelling was immobilized through a biotin-NTA in streptavidin-coated microfluidic chambers for each nucleotide-loading state (Fig. 2b and Supplementary Fig. 7). The FRET of the two fluorophores on each MxA molecule was subsequently recorded, which negatively correlated with the distance between the two labelled sites^33^ (Fig. 2c).

### Conformational preference for GD-BSE region

**Figure 3:**
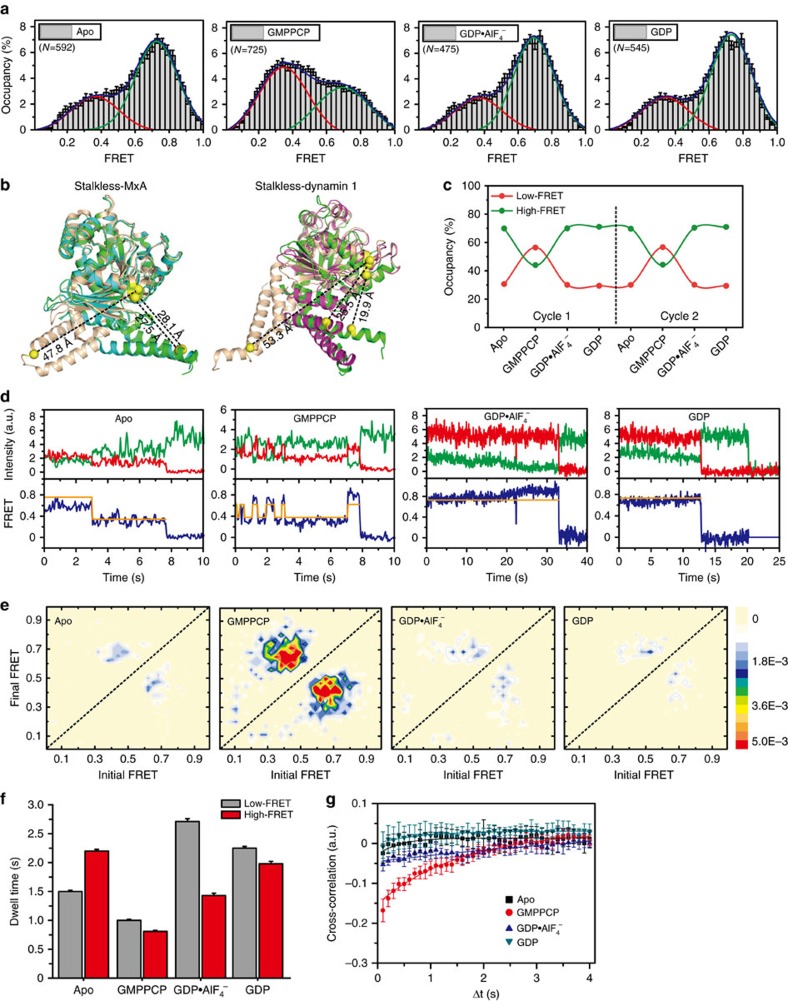
Conformational dynamics in the GD-BSE region. (**a**) Histograms of FRET data from the mutant MxA^MO2^-AS in different nucleotide-loading conditions. Error bars indicate s.d. of 1,000 bootstrap samples of the FRET traces. Decomposition of the FRET data resulted in two Gaussian curves representing distributions of a low-FRET state (red) and a high-FRET state (green). The number of molecules (N) of smFRET trajectories used to construct each histogram is displayed. (**b**) Crystal structures of stalkless MxA (left) and dynamin-1 (right) showing the corresponding distances in different nucleotide-loading forms. For MxA, apo (4P4U, green), GMPPCP-bound (4P4S, wheat) and GDP-bound (4P4T, cyan) forms are shown, and distances between amino acid residues 149 and 639 (634 is missing in these structural models) were measured. For dynamin, apo (3SNH, green), GMPPCP-bound (3ZYC, wheat), GDP·AlF_4_^−^-bound (2X2E, magenta) forms are shown, distances between residues 107 and 726 were measured according to the sequence alignment with MxA. (**c**) Fraction variation in the low-FRET open state (red circle) and the high-FRET closed state (green circle) during GTP hydrolysis. Two GTP hydrolysis cycles are depicted. (**d**) Representative fluorescence and FRET time traces with idealization (orange) in corresponding nucleotide-loading conditions. Here, FRET traces were idealized to a model containing four states corresponding to Gaussian decomposition shown in **a**. (**e**) TDPs in different nucleotide-loading conditions. Initial and final FRET (average FRET values before and after each transition respectively) were plotted as a 2D chart to indicate the transitions between the two distinct FRET states (scale at right). (**f**) Average dwell times in each FRET state in different nucleotide-loading conditions. Error bars indicate s.d. of 100 bootstrap samples. (**g**) Cross-correlation analysis shows enhanced dynamics for GMPPCP-loading condition while limited dynamics for the other three conditions. Error bars indicate s.d. of results from five independent experiments. 2D, two-dimensional; TDP, transition density plot.

We first inspected the relative movement between GD and BSE at different stages of GTP hydrolysis by monitoring the corresponding FRET values for labelled MxA^MO2^-AS at a time resolution of 50 ms. Surprisingly, MxA^MO2^-AS displayed two steadily distinguished FRET states (∼0.35/low and ∼0.72/high, Fig. 3a and Supplementary Fig. 8a). Prior structural studies identified two GD-BSE conformations: MxA/dynamin molecules in apo, GDP·AlF_4_^−^-bound and GDP-bound forms display a closed conformation, while MxA/dynamin bound with non-hydrolysable GTP analogue guanosine-5′-[(β,γ)-methyleno]triphosphate (GMPPCP) adopts an open conformation where the BSE swings ∼70° against the GD^17^,^28^,^29^,^30^ (Fig. 3b). For these two conformations, the distances between A149 and S634 (that is, the fluorophore pair) differed by ∼20 Å, which is in agreement with the margin between the high- and low-FRET values (Fig. 3a). Thus, the high- and low-FRET states represent the closed and open conformations of stalkless-MxA, respectively. The FRET traces in apo, GDP·AlF_4_^−^-bound and GDP-bound forms are very similar and the high-FRET state is dominant with an occupancy of ∼70%, whereas loading of GMPPCP shifts the FRET distribution towards the low-FRET state (Fig. 3c). These results demonstrate that the GMPPCP-dependent BSE rearrangement observed in stalkless-MxA structures also occurs in full-length MxA. More importantly, instead of staying uniform at each nucleotide-loading state, MxA always exhibits two conformations in the GD-BSE region throughout the GTP hydrolysis cycle, albeit with apparent preferences.

### Conformational switch of GD-BSE region

To understand the dynamic rearrangement of the GD-BSE region during the GTP turnover cycle, we analysed FRET trajectories for all imaged MxA^MO2^-AS molecules using hidden Markov modelling. Interestingly, for GMPPCP-bound MxA^MO2^-AS, frequent transitions between the high- and low-FRET states were observed (Fig. 3d,e), indicating that these molecules experienced multiple rounds of switching between the closed and open conformations. The estimated average dwell times in high- and low-FRET states are 0.8 and 1.0 s, respectively (Fig. 3f). In contrast, the conformational transitions are rare in apo, GDP·AlF_4_^−^-bound and GDP-bound conditions (Fig. 3e). The corresponding average dwell times in these conditions are all greater than in the GMPPCP-bound condition, suggesting less frequent domain rearrangement of the molecule (Fig. 3f). We also quantified the structural fluctuations with cross-correlation analyses (Fig. 3g). The cross-correlation amplitude of donor and acceptor signals in GMPPCP are much higher than in the apo condition, suggesting that GMPPCP binding promotes flexibility in the GD-BSE region of MxA^MO2^-AS, which is consistent with transition density plots analysis (Fig. 3e). Anti-correlations between donor and acceptor were almost abolished in the GDP·AlF_4_^−^- and GDP-bound conditions (Fig. 3g), suggesting that the GD-BSE region becomes stabilized after GTP hydrolysis.

### Domain rearrangement in the BSE-stalk region

**Figure 4:**
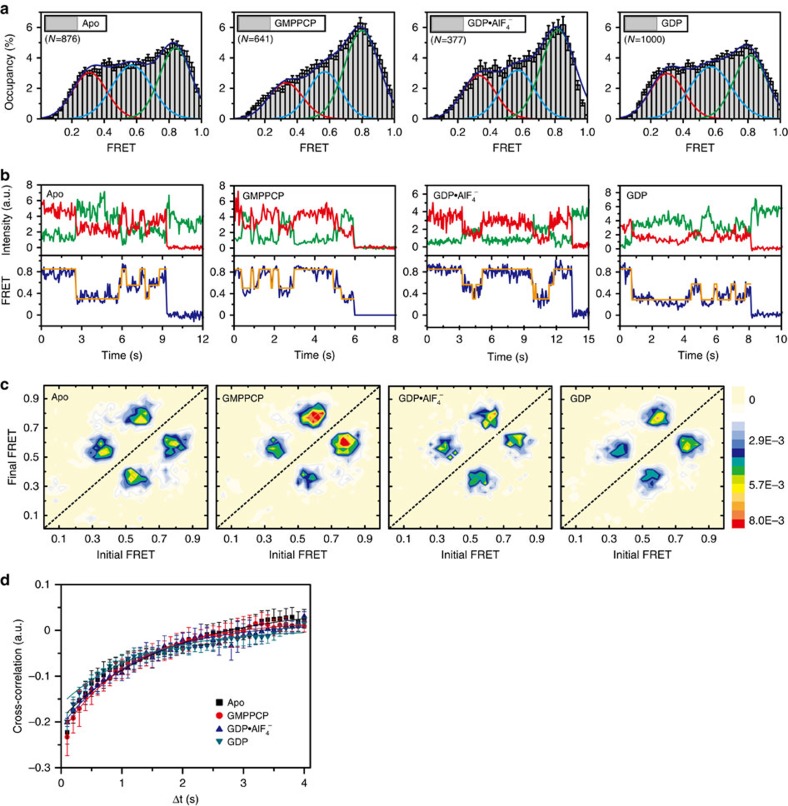
Conformational dynamics in the BSE-stalk region. (**a**) Histograms of FRET data from the mutant MxA^MO2^-RR. Error bars indicate s.d. of 1,000 bootstrap samples of the FRET traces. Gaussian curves for low-, medium- and high-FRET states are coloured red, marine and green, respectively. (**b**) Representative traces for the mutant MxA^MO2^-RR in different nucleotide-loading conditions. Traces and idealization are coloured as in Fig. 3d. (**c**) TDPs as in Fig. 4a (different nucleotide-loading conditions are indicated). (**d**) Cross-correlation analysis shows similar dynamics in different nucleotide-loading conditions. Error bars indicate s.d. of results from five independent experiments. TDP, transition density plot.

To clarify the relative movement between BSE and stalk, we investigated the FRET distributions of labelled MxA^MO2^-RR in different nucleotide-loading conditions. Sites 352 and 500 span 35.3 and 35.6 Å in crystal structures of apo MxA^MO2^ and MxA^FL^, respectively. Despite these values from static models, we observed very broad FRET distributions of MxA^MO2^-RR throughout the GTP hydrolysis cycle, which may be categorized into three states (∼0.35/low, ∼0.55/medium and ∼0.85/high) (Fig. 4a,b, Supplementary Fig. 8b and Supplementary Tables 2 and 3). Compared to apo form, the incorporation of GMPPCP leads to a prominent preferential alternation towards the high-FRET state while against the low-FRET states (Fig. 4a). For the GDP·AlF_4_^−^-loading form, the high-FRET distribution is reminiscent of GMPPCP-bound MxA^MO2^-RR, but the preference for low-FRET states recovers to the equivalent level of that in the apo form. Again, GDP-bound MxA^MO2^-RR behaves similar to the apo form (Fig. 4a,b).

We quantified the structural fluctuations during the GTP hydrolysis cycle. Transition density plots revealed different styles of the FRET transition in these three cases: in apo, GDP·AlF_4_^−^-bound and GDP-bound forms, the low-medium and medium-high mutual FRET transitions occur with similar frequency; whereas GMPPCP-bound MxA^MO2^-RR favours medium-high transitions (Fig. 4c). Moreover, for all trajectories, FRET transition directly between low- and high-FRET states are rarely observed (Fig. 4c). Cross-correlation analysis showed that the BSE-stalk region of MxA^MO2^-RR is conformationally flexible in all conditions (Fig. 4d). Given the broad FRET distribution and frequent FRET transition, we conclude that for MxA^MO2^-RR, the BSE and stalk are engaged in a constant relative movement throughout the GTP turnover cycle, rather than remaining stationary to one another.

### A bracket supports the BSE and hinge 1

**Figure 5:**
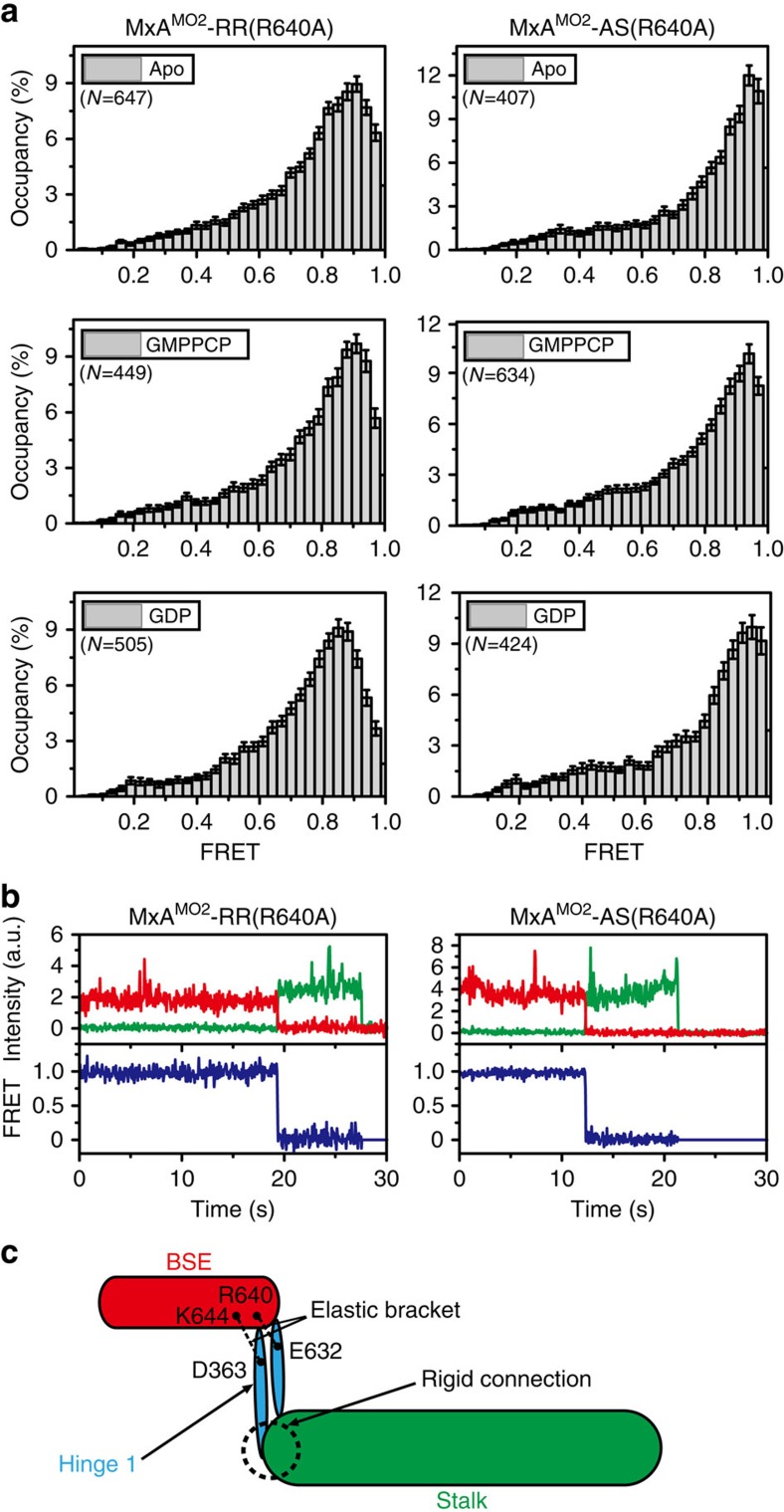
R640A abolishes the flexibility of MxA. (**a**) Histograms of FRET data of MxA^MO2^-RR(R640A) (left) and MxA^MO2^-AS(R640A) (right) in different nucleotide-loading conditions. Error bars indicate s.d. of 1,000 bootstrap samples of the FRET traces. (**b**) Representative traces coloured as in Fig. 2c for MxA^MO2^-RR(R640A) (left) and MxA^MO2^-AS(R640A) (right) in apo condition. (**c**) Schematic drawing of the dual-salt-bridge bracket between the BSE and hinge 1.

Hinge 1 links BSE and stalk, and was thought to be crucial for the function of MxA probably through mediating the BSE-stalk conformation^17^. To explore the exact role of hinge 1, we generated an antiviral negative R640A mutation^17^ on MxA^MO2^-RR and performed smFRET assays on this mutant. In apo, GMPPCP-bound and GDP-bound forms, MxA^MO2^-RR(R640A) displays a single high-FRET state (∼0.9) (Fig. 5a and Supplementary Fig. 8c), indicating a dramatically decreased distance between site 352 and 500. We further introduced R640A mutation to MxA^MO2^-AS and examined it by smFRET assays. Strikingly, MxA^MO2^-AS(R640A) also showed a high-FRET distribution (∼0.95). These extraordinarily high values were not caused by protein aggregation because the FRET trajectories of the corresponding data points all exhibit typical single-molecule features (Fig. 5b). With more than 80% labelling efficiency, we did not observe multiple photobleaching steps for Cy3 or Cy5 (Fig. 5b). Therefore, these results implicate that the domains of MxA^MO2^-AS(R640A) and MxA^MO2^-RR(R640A) become tightly packed and the molecules lose flexibility.

To explain this result, we reviewed the crystal structure of MxA^MO1^ at the corresponding region. Hinge 1 is substantially attached to the proximal end of the stalk via a hydrophobic network which allows very limited relative movement (Figs 1b and 5c). On the other side, salt bridge pairs involving D363-R640 and E632-K644 from BSE and hinge 1 may act as a key bracket that supports the BSE from hinge 1 while allowing a certain level of elasticity (Figs 1b and 5c). Thus, mutation of the central R640 would engender the collapse of this bracket, hence the structural defects of MxA.

## Discussion

**Figure 6:**
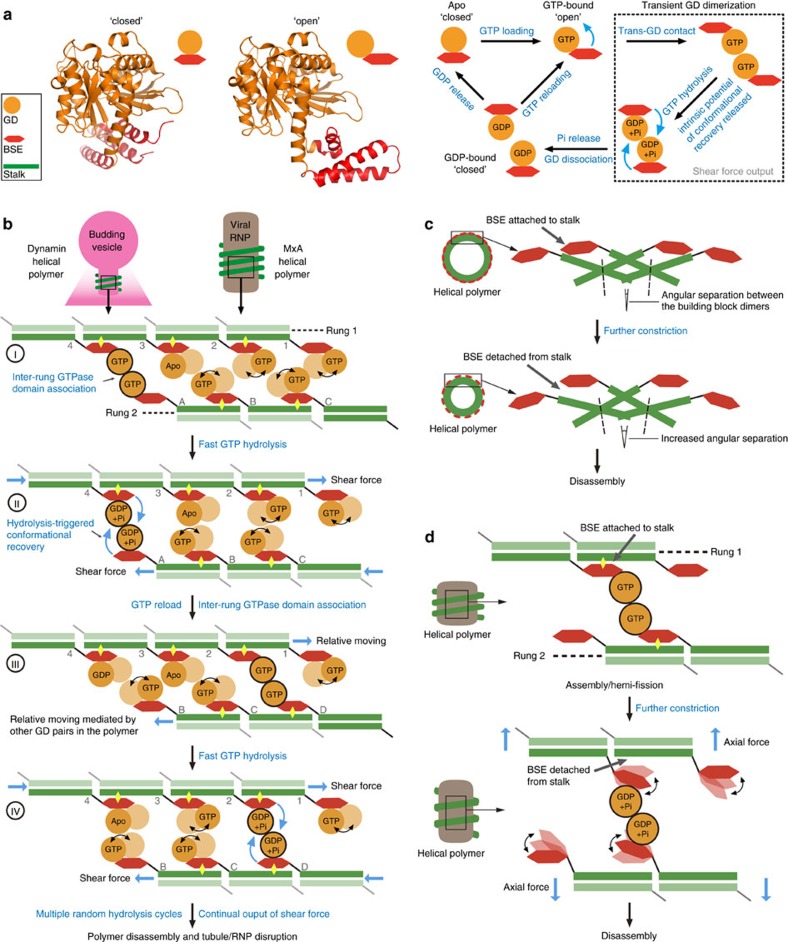
Proposed mechanism for polymer constriction and disassembly. (**a**) Schematic representation of the process of shear force output in the GTP hydrolysis cycle. GDs and BSEs are coloured as in Fig. 1a, and stalks are omitted for clarity. (**b**) Random transient GD interactions and subsequent GTP hydrolysis-dependent conformational changes in the GD-BSE region collectively drive the twisting/constriction of the MxA/dynamin polymer. Two neighbouring rungs are shown as two linear oligomers. The opposing molecules from upper and lower oligomers are numbered 1–4 and A–D, respectively. The BSE-stalk interactions are indicated by yellow diamonds. (I) Loading of GTP promotes domain movement. The GDs of molecule 4 and A associate at open conformation. (II) Stimulated GTP hydrolysis releases the potential of conformational alternation in the GD-BSE region of molecule 4 and A, and the GDs move back to closed conformation, thereby generating latitudinal shear force. Other isolated GDs do not interfere with the relative movement of neighbouring rungs. (III) The shear force leads to the relative movement of the two rungs. Molecule 4 and A may be quickly reloaded with GTP, and the GTP-loaded GDs may randomly form inter-rung contact again, as for molecule 2 and D. (IV) Stimulated GTP hydrolysis occurs and shear force is continually generated as in (II). (**c**) Possible mechanism for the release of BSEs from the neighbouring stalks. The MxA/dynamin helical polymer are shown in an intersection view. Constriction leads to decreased number of molecules in a helical rung and increased angular separation between the neighbouring building block dimers, which causes detachment of BSEs and stalks. GDs are omitted for clarity. (**d**) Schematic representation showing the proposed process of axial force generation. Detachment of BSEs from neighbouring stalks release the conformational flexibility of the BSE-stalk region. As stalks are still associated together, movement of BSEs will generate mechanic forces that may include an axial fraction. More GTP turnover cycles can boost the axial force output that eventually pushes the neighbouring rungs away from each other. In this case, the helical MxA/dynamin polymer exhibits a ‘poppase' feature^43^. Helical polymers are depicted as green spirals around brown substrates. Part of the GDs and BSEs are omitted for clarity.

Prior studies suggested a mechanistic model for MxA and dynamin involving the generation of latitudinal shear force or powerstroke between neighbouring polymer rungs, through a GTP-loading/hydrolysis-regulated rearrangement of the GD-BSE region^26^,^29^,^31^,^40^. Our smFRET results partly confirm this model and provide new insight into full-length MxA by presenting the evidence that MxA is conformationally dynamic in solution. Such dynamics are influenced by, but not absolutely dependent on, nucleotide-loading states. Given the structural similarity to MxA, dynamin may also adopt this feature. A key step, for the majority of the molecules of a functioning polymer, is that GTP hydrolysis triggers the alternation of the conformational preference. This in turn releases the intrinsic potential for conformational recovery, thereby producing a mechanistic force that drives the transition from ‘open' to ‘closed' conformation at the GD-BSE region (Figs 3c and 6a). Importantly, our FRET measurement for MxA^MO2^-AS in GDP·AlF_4_^−^-bound form was designed in such a way that the signals were recorded only for the GD-mediated dimer (termed *trans*-G-dimer, Supplementary Fig. 7, see also Methods), indicating that the open-to-closed conformational transition is completed before the *trans*-G-dimers dissociate. For the MxA/dynamin polymer, the hydrolysis-induced mechanistic force at the GD-BSE region can thus be transformed to the shear force between neighbouring rungs.

A subsequent question is: how are MxA/dynamin molecules regulated to continually output such shear force? In the physiological environment, it is not likely that all component molecules of an MxA/dynamin polymer are always synchronized during GTP hydrolysis, as this would result in intermittent but not the observed smooth constriction of the lipid tubule during multiple rounds of GTP hydrolysis^31^,^41^. On the basis of the smFRET results, we propose a mechanistic model to address this issue. Once the helical polymers are formed around proper substrates, individual MxA/dynamin molecules may fall in any one of the nucleotide-loading states depending on the catalytic stage and local concentrations of guanine nucleotides, and therefore their GD-BSE region may stay at either an open or closed conformation with corresponding preferences (Figs 3a and 6b). GTP-loaded molecules from neighbouring rungs associate with each other in a random manner and generate shear force upon hydrolysis as previously described (Fig. 6a,b). Activated GD movement relative to BSE, as reflected by GMPPCP-bound MxA^MO2^-AS in FRET transition analysis (Fig. 3d), may increase the chance of in *trans* contact of GDs. Moreover, the period of GD dimerization during catalysis was demonstrated to be transient^29^, which is in agreement with the fast stimulated GTP turnovers of dynamin^42^,^43^ and MxA^25^. Transient GD dimerization ensures the smooth relative movement between the rungs and promotes the efficiency of force transmission (Fig. 6b).

Considering their higher binding affinity to GTP over GDP^43^,^44^,^45^ and the negligible intrinsic GTP turnover^42^, MxA/dynamin molecules after hydrolysis may be promptly reloaded with GTP. As a result, random *trans*-GD-interactions can occur frequently and repeatedly between molecules of neighbouring rungs, collectively producing a continual output of latitudinal shear force that is statistically favoured in the same direction (Figs 3a and 6b). Propagation of this process generates a stable torque that twists the polymer helix regardless of the variation of the molecule numbers in the rungs. For dynamin, such torque will lead to a gradual constriction of the lipid tubule until it is ruptured or the hemi-fission state is achieved^31^,^41^. According to our model, as long as one complete helical turn of dynamin/MxA polymer is formed, the aforementioned conformational transitions occurring on the first inter-rung pair of molecules may be sufficient to initiate the constriction event. This is in agreement with the fact that dynamin typically forms no more than one helical turn around the neck of budding vesicles in living cells^46^,^47^. In addition, based on the FRET distributions, there can be a minority of molecules in a polymer that do not perform hydrolysis-dependent open-to-closed conformational change (Fig. 3a). These molecules may undergo futile GTP turnovers that delay constriction, which partly explains the relatively long lifetime (4–6 s), with respect to the high stimulated GTP turnover rate of assembled dynamin polymer, during the endocytosis process^46^.

Before the MxA/dynamin polymer disassembles, the relative movement between the BSE and stalk in the same molecule is likely to be restrained by intermolecular BSE-stalk associations^17^,^26^ (Fig. 6c). For dynamin, it was suggested that complete fission reaction needs a hydrolysis-related energy input, which probably produces axial force that is in coordination with the disassembly of the polymer scaffold^31^. A possible event related to this input might be the detachment of BSE from neighbouring stalk on the same rung, presumably caused by the increasing angular separation between the building block dimers during the progression of constriction (Fig. 6c). Consequently, the released potential of conformational dynamics will lead to vigorous relative movement in the stalk-BSE region. Such movement may generate mechano-forces with an axial fraction that eventually destabilize the polymer scaffold and facilitate the functional disassembly of the polymer (Fig. 6d). The movements of PH domain were also suggested to regulate dynamin-mediated membrane fission^31^,^32^. For MxA and dynamin-1-like protein (DNM1L) which lack the PH domain, the conformational dynamics of the BSE-stalk region may play a crucial role in the disassembly of helical polymers^23^,^48^.

## Methods

### Protein expression and purification

cDNAs of all human MxA constructs, including those for crystallization and smFRET assay were individually cloned into a modified pET28 vector. Details of these constructs were summarized and illustrated in Supplementary Fig. 1a. For these constructs, recombinant proteins containing an N-terminal His_6_-tag followed by a cleavage site for PreScission protease (PSP) were expressed in *Escherichia coli Rosetta* (DE3) cells. Transformed bacteria were cultured at 37 °C before induced with 0.1 mM isopropyl-1-thio-β-D-galactopyranoside (IPTG) at an OD_600_ of 0.6, and grown overnight at 18 °C. Cells were lysed in 50 mM HEPES (pH 7.3), 400 mM NaCl, 30 mM imidazole, 6 mM MgCl_2_, 1 μM DNase, 1 mM Phenylmethanesulfonyl fluoride (PMSF), 2.5 mM β-mercaptoethanol (β-ME) at 4 °C and kept at this temperature all through the following purification steps. After centrifugation at 40,000 *g* for 50 min, the supernatant was filtered and applied to a Ni-NTA column (GE Healthcare) equilibrated with 50 mM HEPES (pH 7.3), 400 mM NaCl, 30 mM imidazole, 5 mM MgCl_2_, 2.5 mM β-ME. The column was extensively washed with 20 mM HEPES (pH 7.3), 800 mM NaCl, 5 mM MgCl_2_, 30 mM imidazole, 2.5 mM β-ME, 1 mM ATP, 10 mM KCl and shortly with 20 mM HEPES (pH 7.3), 400 mM NaCl, 5 mM MgCl_2_, 80 mM imidazole and 2.5 mM β-ME. Proteins were subsequently eluted with 20 mM HEPES (pH 7.3), 400 mM NaCl, 300 mM imidazole, 5 mM MgCl_2_, 2.5 mM β-ME and dialysed overnight against 20 mM HEPES (pH 7.3), 400 mM NaCl, 4 mM MgCl_2_, 2.5 mM β-ME (Dialysis Buffer) in the presence of 10 μg PSP to remove the His_6_-tag. After dialysis, PSP was removed using a GST column, and proteins were reapplied to a Ni-NTA column equilibrated with Dialysis Buffer. After short wash with dialysis buffer, the proteins were eluted with 20 mM HEPES (pH 7.3), 400 mM NaCl, 30 mM imidazole, 4 mM MgCl_2_, 2.5 mM β-ME. The proteins were further purified using size-exclusion chromatography on a Superdex200 26/60 column (GE Healthcare) equilibrated with 20 mM HEPES (pH 7.3), 150 mM NaCl, 4 mM MgCl_2_, plus either 1 mM dithiothreitol (DTT) for MxA^MO1^ (construct for crystallization) or 1 mM Tris(2-carboxyethyl)phosphine (TCEP) for MxA^MO2^, MxA^MO2^-AS, MxA^MO2^-RR, MxA^MO2^-AS^R640A^ and MxA^MO2^-RR^R640A^ (constructs for smFRET assay). Where they eluted in a discrete peak at ∼70 kDa. In addition, for all constructs for smFRET assay, another N-terminally His_6_-tagged edition was purified by Ni-NTA affinity chromatography and SEC as described above, excluding the steps of dialysis, PSP digestion and reapplication to Ni-NTA.

### Crystallization and structure determination

MxA^MO1^ Crystals were obtained using hanging-drop vapour diffusion method by mixing 1 μl protein (18 mg ml^−1^) with an equal volume of reservoir solution containing 100 mM ammonium citrate tribasic (pH 7.0) and 12% PEG3350 at 18 °C. A cryo-solution containing 6% PEG3350, 50 mM ammonium citrate tribasic (pH 7.0), 15% glycerol was used for flash-cooling the crystals in liquid nitrogen. The data set was collected from a single crystal at beamline BL17U1 of the Shanghai Synchrotron Radiation Facility and processed using the XDS program suite^49^. The phase problem was solved by molecular replacement using MolRep^50^ with the atomic coordinates of nucleotide-free stalkless-MxA (4P4U)^29^ and MxA (3LJB)^25^ as search models. Models were built with COOT^51^ and refined with Refmac^52^ and Phenix^53^. Structural validation was carried out using MolProbity^54^. Structural illustrations were prepared using the PyMOL Molecular Graphics Systems (version 0.99, Schrödinger LLC; http://www.pymol.org/). The Ramachandran statistics of the MxA^MO1^ structure determined by PROCHECK^55^ are: 97.02% in favoured region, 2.28% allowed, 0.69% outlier.

### Dimerization assay

To measure the absolute molecular mass of MxA^MO1^ in solution, a coupled right-angle light-scattering-refractive index detector (Malvern) was connected in line to a Superdex200 10/300 SEC column. 100 μM purified MxA^MO1^ was applied to the column equilibrated with 20 mM HEPES (pH 7.3), 150 mM NaCl, 4 mM MgCl_2_, 1 mM DTT. Data were analysed with the provided OMNISEC software. For MxA^MO2^-AS, MxA^MO2^-RR, 50 μM purified proteins were incubated with or without 1 mM GDP, 1 mM AlCl_3_ and 10 mM NaF for 3 h at room temperature prior to the SEC assay in the same buffer. All experiments were repeated at least twice and the data showed satisfying consistency.

### GTP hydrolysis assay

The GTP turnover rates of MxA^MO2^-AS and MxA^MO2^-RR were determined as described earlier^25^. Briefly, 20 μM protein was incubated with 4 mM GTP at 37 °C in 20 mM HEPES (pH 7.3), 150 mM NaCl and 4 mM MgCl_2_, 1 mM DTT. At different time points, reaction aliquots were acquainted and the GTP:GDP ratios were measured using a high performance liquid chromatography-based method, and the initial rates of the reaction (<40% GTP hydrolysed) were thereby calculated.

### Nucleotide-binding studies

The equilibrium dissociation constants for MxA^MO2^-AS and MxA^MO2^-RR to GDP and GMPPCP were determined by isothermal titration calorimetry using a MicroCal ITC200 (Malvern) at 25 °C. 1.5 mM nucleotide was titrated at 2 μl step against 80 μM protein in the buffer containing 20 mM HEPES (pH 7.3), 150 mM NaCl, 4 mM MgCl_2_. Resulting heat change upon each injection was integrated and the values were fitted to a standard single-site binding model using Origin7. All experiments were repeated at least twice and the *K*_d_ values derived from the experimental replicates were consistent with corresponding results shown in the paper.

### Dye labelling

Corresponding MxA^MO2^ derivatives were labelled with Cy3- and Cy5-maleimide dyes^56^ (GE Healthcare), as donor and acceptor respectively. For labelling, the protein was incubated with a fivefold molar excess of respective dye for 1 h at 4 °C in the buffer containing 20 mM HEPES (pH 7.0), 150 mM NaCl, 4 mM MgCl_2_ and 0.5 mM TCEP. The reaction was quenched by the addition of L-cysteine with 100-fold molar excess of two dyes and then the excess free dyes were removed using Zeba spin desalting columns (Thermo Scientific). In addition, for imaging experiments on dimeric GDP·AlF_4_^−^-bound proteins, the samples were prepared as described in Supplementary Fig. 5. Dye-labelled protein without His_6_-tag was incubated with His_6_-tagged protein which was not labelled (yellow) in the presence of 1 mM GDP, 1 mM AlCl_3_ and 10 mM NaF. These proteins were subsequently applied to SEC and the fractions of the dimers were collected for immobilization.

### Steady-state fluorescence anisotropy measurements

Steady-state anisotropy measurements were carried out using a PTl spectrofluorometer with excitation and emission wavelengths of 532 and 560 nm, respectively. A total of 100 nM of Cy3-labelled corresponding MxA constructs was measured in 20 mM HEPES (pH 7.0), 150 mM NaCl, 4 mM MgCl_2_ and 0.5 mM TCEP as described in Supplementary Fig. 6b.

### Single-molecule FRET experiments

All single-molecule FRET measurements were performed in buffer containing 20 mM HEPES (pH 7.3), 150 mM NaCl, 4 mM MgCl_2_ and 0.5 mM TCEP at room temperature. An oxygen-scavenging environment (1 unit per ml glucose oxidase, 8 units per ml catalase, 0.1% v/v glucose) containing 1 mM cyclooctatetraene was used in all experiments to minimize photobleaching^57^. Microfluidic imaging chambers on the quartz slide surface coated with a mixture of PEG and biotin-PEG^58^,^59^ were incubated with 0.8 μM streptavidin (Invitrogen), followed by 20 nM biotin-NTA (Biotium) charged with NiCl_2_ (refs 60, 61), so as to eliminate nonspecific surface adsorption of the protein. The immobilization of corresponding Cy3/Cy5-labelled His_6_-tagged proteins (5 nM) was mediated by surfaced-bound Ni^2+^. Next, 100 μM desired nucleotides was directly added and incubated with the surface-tethered molecules of corresponding MxA^MO2^ derivatives for 10 min before data acquisition. Fluorescence experiments were performed using an objective based total internal reflection fluorescence microscope. Cy3 fluorophore was excited with 532 nm laser (Coherent Inc, Sapphire SF). Photon emitted from Cy3 and Cy5 were collected using 1.49 NA 100 × objective (Olympus UPAPON 100 × OTIRF), and Optosplit II(Cairn Research Limited) was used to separate spatially Cy3 and Cy5 frequencies onto a cooled EMCCD (Andor iXon Ultra). Fluorescence data were acquired using the software Metamorph (Universal Imaging Corporation). Single-molecule FRET-time traces were recorded with a time resolution of 50 ms and FRET traces were calculated as: FRET=*I*_Cy5_/(*I*_Cy3_+*I*_Cy5_), where *I*_Cy3_ and *I*_Cy5_ are the instantaneous Cy3 and Cy5 fluorescence intensities, respectively^61^,^62^. Cy3 and Cy5 channel were mapped using tetraSpeck fluorescent microsphere beads (Invitrogen, 0.1 μm). At least more than 10 beads were selected to get the transformation matrix used in the mapping in Matlab. Photobleaching events in each traces were detected as a significant drop (≥3 times s.d. of background noise) in the median filtered (window size=9 frames) total fluorescence intensity (*I*_total_=*I*_cy3_+*I*_cy5_) without returning to the previous average level. Signal-to-background noise ratios are calculated as total intensity relative to the s.d. of background noise: *I*_total_/[stdev(*I*_cy3_)+stdev(*I*_cy5_)]. Traces were selected automatically to meet the following criteria: a single catastrophic photobleaching event, at least 8:1 signal-to-background noise ratio, a donor-to-acceptor Pearson's correlation coefficient<0. Spectral bleed-through of Cy3 intensity on the acceptor channel was corrected by subtracting 7.5% of donor signal from the acceptor. All the data were repeated three times and we did not found significant difference. The MATLAB code for analysing the cross-correlation of donor and acceptor intensity is a gift from Dr Reza Vafabakhsh (University of California, Berkeley)^63^. The cross-correlation calculation was performed on the same traces that were used for the histogram.

### Minireplicon assay

The polymerase activity of influenza A virus, strain A/Vietnam/1203/04 (H5N1)^64^ was reconstituted in 293T cells seeded into 12-well plates and transfected (JetPEI, Polyplus) with 10 ng pCAGGS expression plasmids for the viral polymerase subunits PB2, PB1 and PA as well as 100 ng NP-encoding plasmids. As minigenome, 50 ng of plasmids encoding Firefly luciferase in negative sense orientation flanked by 5′- and 3′-UTRs from viral segment 8 (pPolI-FFLuc-RT) were co-transfected. The cell line is free of mycoplasma. 10 ng pRL-SV40 constitutively expressing Renilla luciferase was added to normalize transfection efficiency. It was shown that expression of the Firefly luciferase reporter gene correlates with the activity of the reconstituted polymerase complex^65^. To examine MxA-mediated inhibition of viral polymerase activity, pCAGGS plasmids encoding N-terminally Flag-tagged MxA mutants were co-transfected. T103A, wt, A149C/S634C, R352C/R500C, C4 (C42S/C52S/C322K/C336Q) and M527D: 300 ng plasmids per well. C4/A149C/S634C and C4/R352C/R500C: 400 ng plasmids per well. M527D/C4, M527D/C4/A149C/S634C and M527D/C4/R352C/R500C: 600 ng plasmids per well. Cells were lysed at 24 h post-transfection and Firefly and Renilla luciferase activities were measured using the dual luciferase reporter assay (Promega). The experiment was performed three times and each experiment contained technical duplicates. After normalizing Firefly to Renilla luciferase activity, one duplicate of the empty vector control was set to 100%. Results are presented as means of technical duplicates of three independent experiments. Western blot analysis with the mouse monoclonal anti-Flag antibody (Sigma Aldrich, clone M2, catalogue number F3165), rabbit polyclonal anti-β-actin antibody (Abcam, ab8227) and mouse monoclonal anti-influenza A virus NP (Bio-Rad, clone AA5H, MCA400) was performed to verify proper expression of the MxA variant.

### Data availability

Atomic coordinates and structure factors for MxA^MO1^ have been deposited with the Protein Data Bank under accession code 5GTM. The data that support the findings of this study are available from the corresponding author upon reasonable request.

## Additional information

**How to cite this article:** Chen, Y. *et al*. Conformational dynamics of dynamin-like MxA revealed by single-molecule FRET. *Nat. Commun.* **8**, 15744 doi: 10.1038/ncomms15744 (2017).

**Publisher's note:** Springer Nature remains neutral with regard to jurisdictional claims in published maps and institutional affiliations.

## Supplementary Material

Supplementary Information Supplementary Figures, Supplementary Tables and Supplementary References
