# Supplementary material for: Conformational dynamics of dynamin-like MxA revealed by single-molecule FRET
Source: Nat Commun. 2017 May 26;8:15744. doi: 10.1038/ncomms15744 (PMC5458555; doi:10.1038/ncomms15744)
Supplement: Supplementary Information — Supplementary Figures, Supplementary Tables and Supplementary References [file ncomms15744-s1.pdf]

Supplementary Information

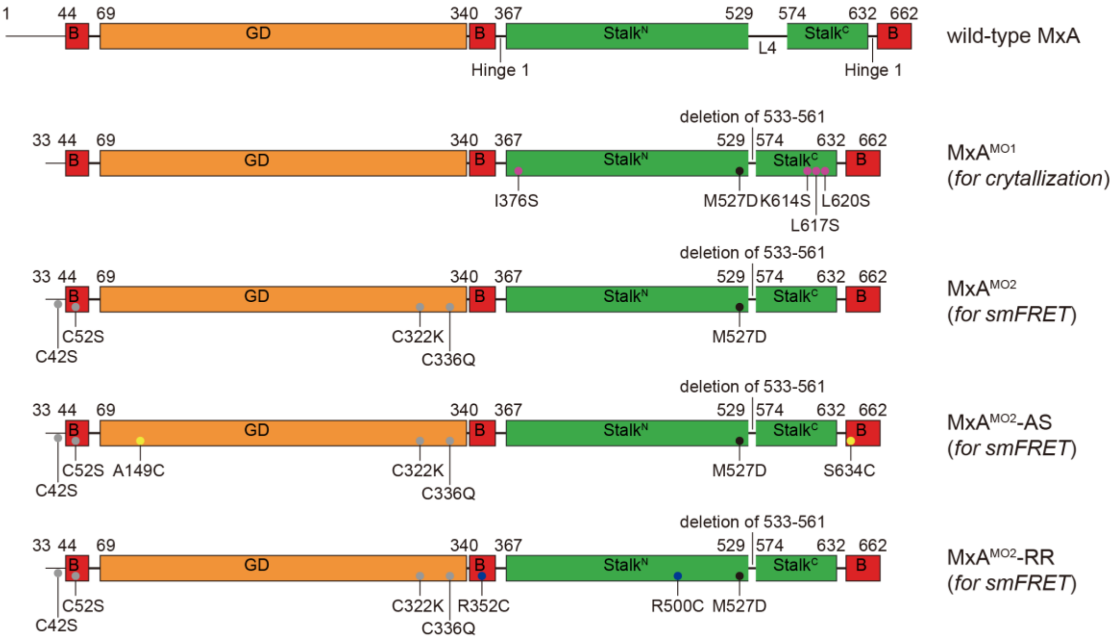

Supplementary Figure 1: Summary of the monomeric MxA constructs.

Schematic representation of the monomeric MxA constructs compared to the wild-type full-length version. Domains are coloured as in Fig. 1**a**. Mutations on each construct are indicated at corresponding positions with filled circles which are coloured as follows: interface 1 mutations in purple, interface 2 mutation in black, inherent cysteine mutations in grey, the GD-BSE FRET-pair mutations in yellow, and the BSE-stalk FRET-pair mutations in blue.

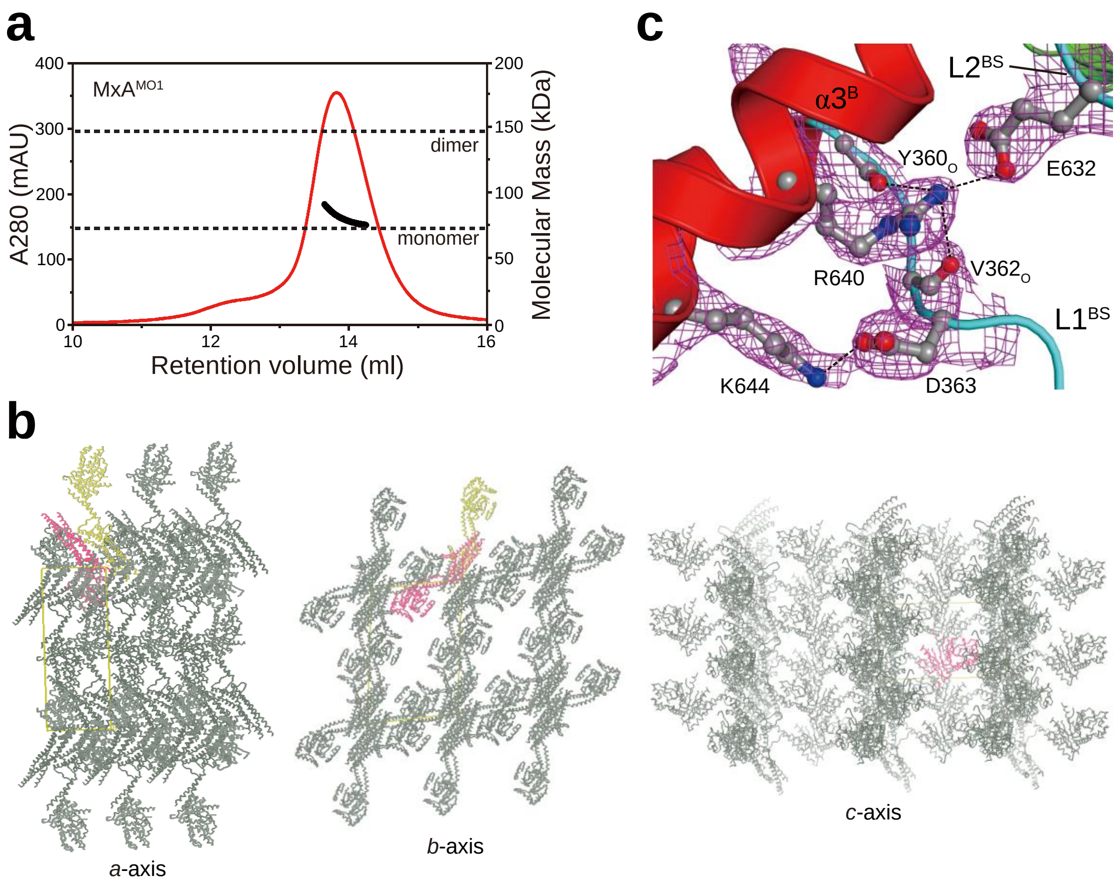

Supplementary Figure 2: The monomeric feature of MxA^{MO1}.

a, Size-exclusion chromatography coupled with right-angle light scattering (SEC-RALS) assay shows that MxA^{MO1} is a monomer in solution. The 280 nm absorbance curve is shown in red, and the calculated molecular mass at the absorption peak is plotted in black. The molecular mass values of monomeric and dimeric MxA^{MO1} are indicated by dashed lines.

b, Crystal packing of MxA^{MO1} viewed along *a*, *b* and *c*-axis of the unit cell. Two MxA^{MO1} molecules are in the asymmetric unit of the crystal. The two chains of a non-crystallographic dimer are coloured in magenta and yellow, respectively, as representatives.

c, Electron density map of residues involved in the interaction between hinge 1 and BSE. Involved residues are shown in ball-and-stick representation and the corresponding electron density is shown at a contour level of 1.2σ as violet mesh.

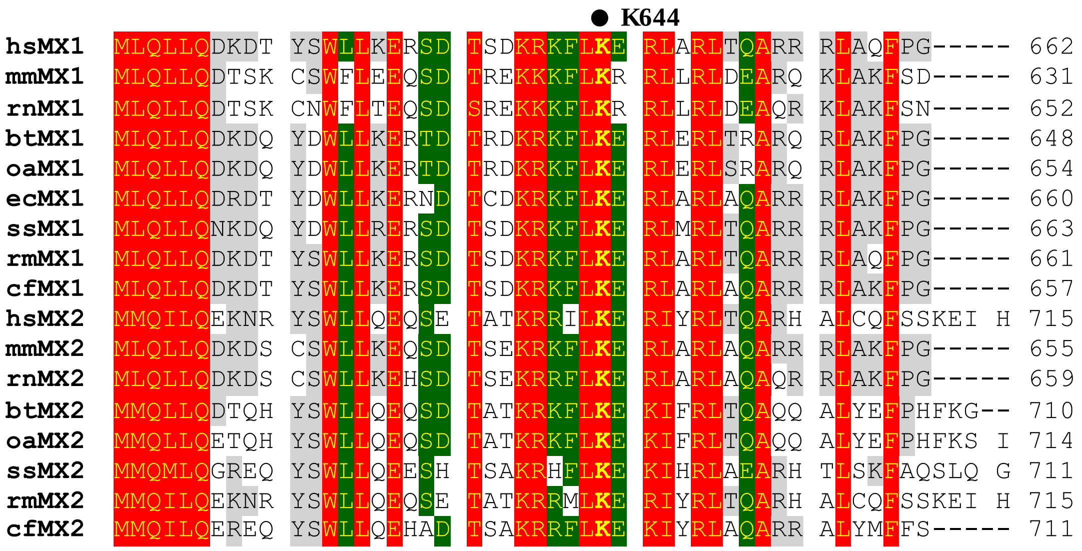

Supplementary Figure 3: Sequence alignment of mammalian Mx proteins.

Sequence alignment of mammalian Mx proteins. Amino acid sequences of human (hs) Mx1 (UniProt accession P20591), mouse (mm) Mx1 (P09922), rat (rn) Mx1 (P18588), bovine (bt) Mx1 (P79135), sheep (oa) Mx1 (P33237), horse (ec) Mx1 (Q28379), pig (ss) Mx1 (P27594), *Rhesus macaque* (rm) Mx1 (A1E2I4), dog (cf) Mx1 (Q9N0Y3), human Mx2(P20592), mouse Mx2 (Q9WVP9), rat Mx2 (P18589), bovine Mx2 (Q9BDI7), sheep Mx2 (Q5I2P5), pig Mx2 (A7VK00), *Rhesus macaque* Mx2 (A1E2I5), dog Mx2 (Q9N0Y2) are aligned using Clustal W[1]. Residues with a conservation of 100% are in red shades, greater than 80% in green shades and 50% in grey shades, respectively. Residue K644 (●) in different Mx proteins is indicated.

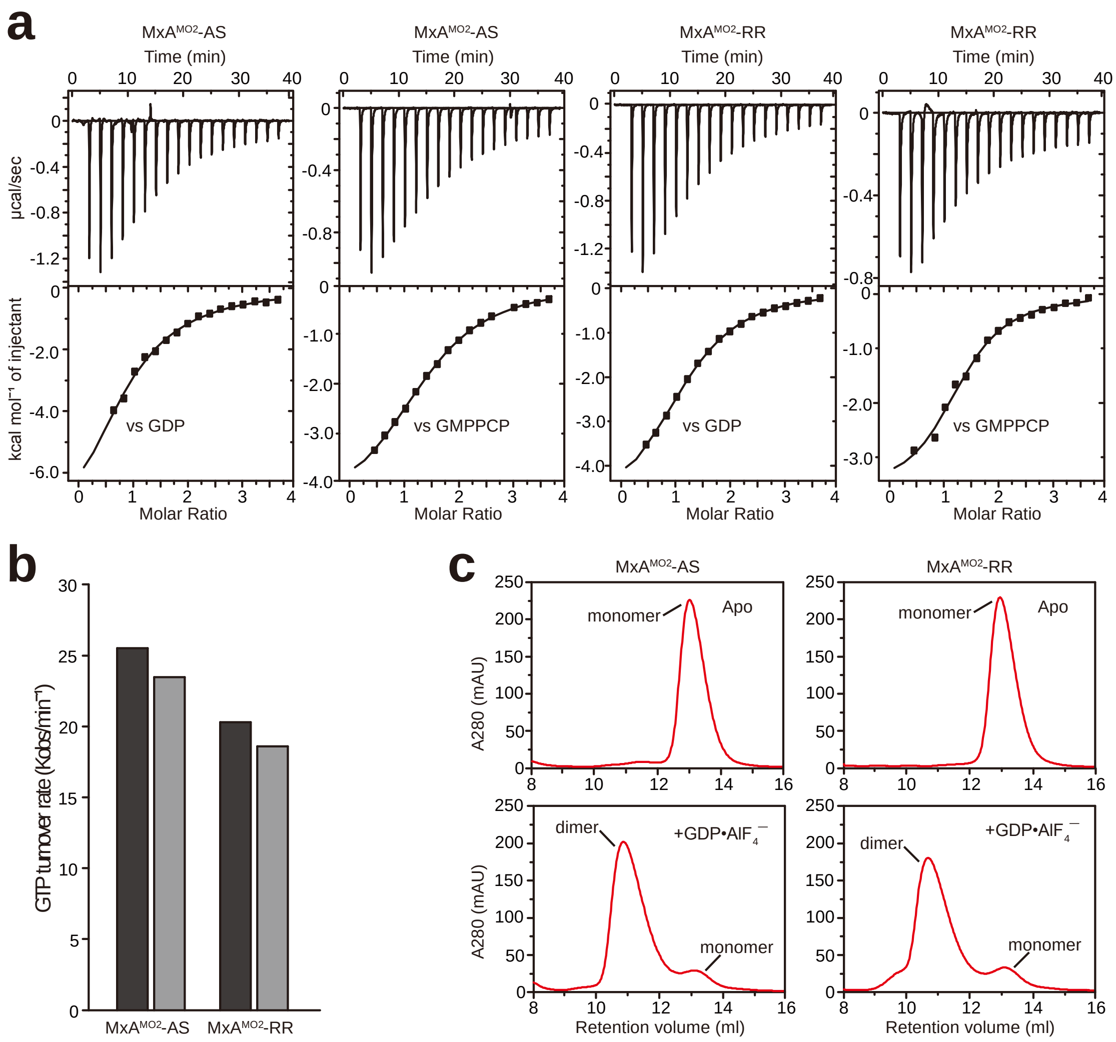

Supplementary Figure 4: Biochemical validation of the FRET-compatible MxA constructs.

a, Binding affinity of MxA^{MO2}-AS and MxA^{MO2}-RR to guanine nucleotides was measured by ITC. The dissociation constants (K_d) and binding sites (N) are: MxA^{MO2}-AS vs GDP: K_d=42.6±0.14 μM, N=0.89±0.12, MxA^{MO2}-AS vs GMPPCP: K_d=30±0.003 μM, N=1.43±0.01, MxA^{MO2}-RR vs GDP: K_d=26.3±0.03 μM, N=1.22±0.01, MxA^{MO2}-RR vs GMPPCP: K_d=14.7±0.13 μM, N=1.31±0.04.

b, GTPase activities of MxA^{MO2}-AS and MxA^{MO2}-RR. Results from two separated experiments are individually presented in black and grey.

c, SEC analysis shows that both MxA^{MO2}-AS (left) and MxA^{MO2}-RR (right) are monomers in the apo condition and can dimerize in the GDP•AlF_4^--loading condition.

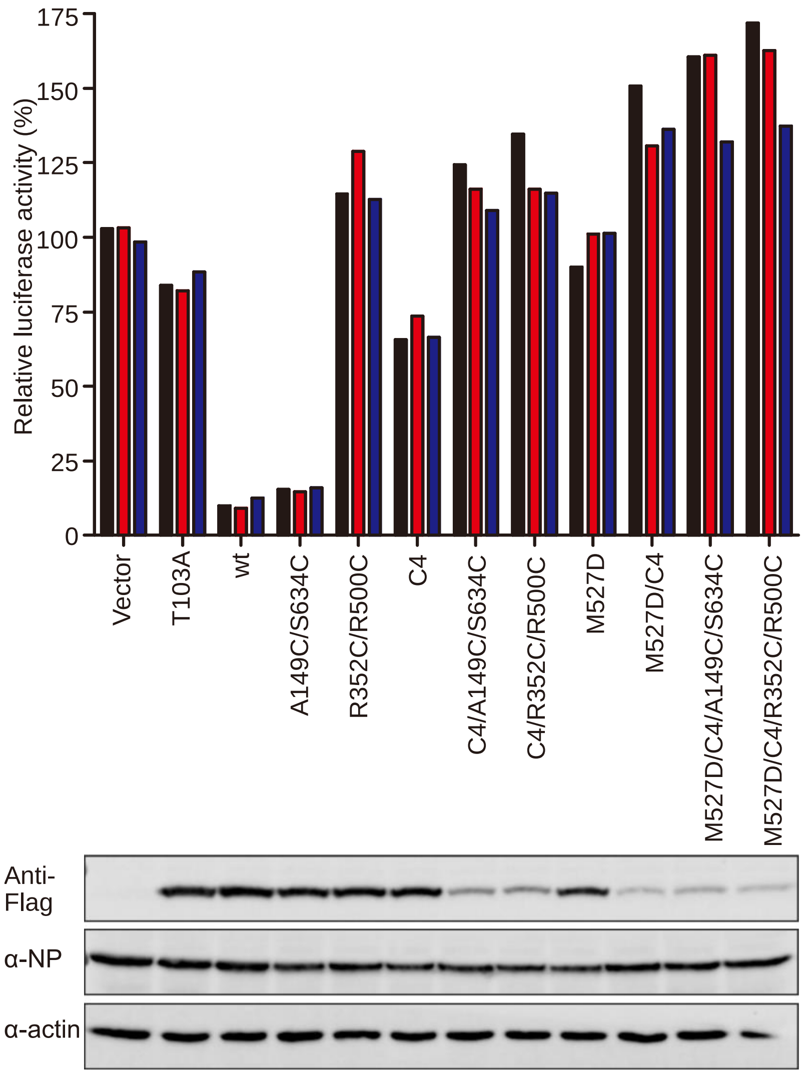

Supplementary Figure 5: Minireplicon assay for smFRET mutants.

Minireplicon assay for influenza A virus (A/Vietnam/1203/04, H5N1) polymerase. 293T cells were transfected with plasmids encoding viral nucleoprotein (NP), the viral polymerase subunits, and a reporter construct encoding firefly luciferase under the control of the viral promoter. Expression plasmids for the indicated MxA constructs and for Renilla luciferase under a constitutive promoter were co-transfected. Twenty-four hours later, the activity of firefly luciferase was measured and normalized to the activity of Renilla luciferase. The empty vector control was set to 100%. Results from three separated experiments are individually presented in black, red and blue for each sample. Protein expression was verified by Western blot analyses using specific antibodies against the Flag-tag, viral NP and actin. C4 denotes the quadruple mutation of C42S, C52S, C322K and C336Q.

a

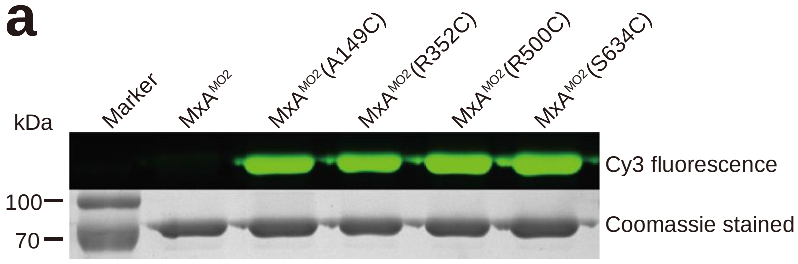

b

	Apo	GDP	GMPPCP	labelling efficiency
cy3-free	0.226±0.001	0.228±0.012	0.229±0.001	N/A
cy3-labelled A149C	0.196±0.002	0.214±0.006	0.194±0.002	80.0%
cy3-labelled S634C	0.240±0.004	0.234±0.009	0.244±0.009	86.0%
cy3-labelled R352C	0.202±0.002	0.201±0.007	0.189±0.001	99.7%
cy3-labelled R500C	0.243±0.021	0.254±0.004	0.248±0.002	82.5%

Supplementary Figure 6: Fluorescence properties of MxA^{MO2} mutants.

a, Site-specific dye-labelling of four single-point mutants based on MxA^{MO2}. MxA^{MO2} and the mutants were incubated with Cy3-maleimide as described in **Methods**. The SDS-PAGE results showed that the mutants were efficiently labelled as indicated by high fluorescence. In contrast, MxA^{MO2}, which has no exposed cysteines, emitted no fluorescence. The same gel stained with Coomassie brilliant blue is shown below.

b, Fluorescence anisotropy analysis and labelling efficiency of each mutation site. Fluorescence anisotropy was measured for Cy3-free and four Cy3-labelled single-point mutants in solution containing 20 mM HEPES (pH 7.0), 150 mM NaCl, 4 mM $MgCl_2$ and 0.5 mM TCEP. Each measured anisotropy value is shown as mean±standard deviation. Labelling efficiency was calculated as [Cy3]/[protein] ([Cy3] and [protein] denote the molar concentrations of Cy3 or protein for each Cy3-labelled single-mutant, respectively).

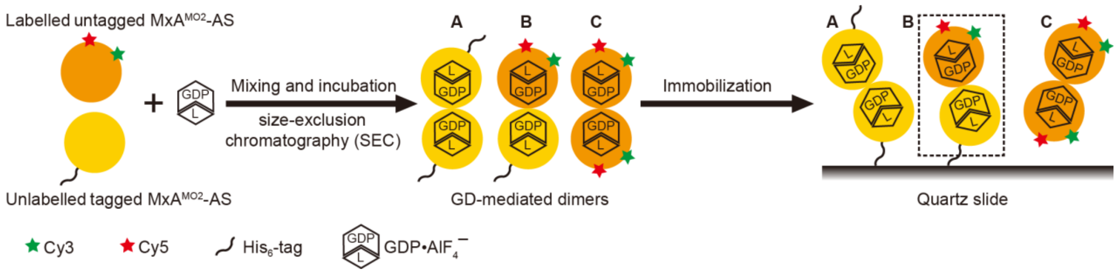

Supplementary Figure 7: Immobilization of dimeric GDP•AlF_4^--bound MxA.

In order to measure the FRET of GDP•AlF_4^--bound MxA in the form of GD-mediated dimer, we designed a protocol to immobilize heterodimers that contain only one dye-labelled MxA^{MO2}-AS or MxA^{MO2}-RR as illustrated in this figure. Here, MxA^{MO2}-AS is taken as an example: first, dye-labelled MxA^{MO2}-AS without His_6-tag (orange) was incubated with His_6-tagged MxA^{MO2}-AS which was not labelled (yellow) in the presence of GDP•AlF_4^- (realized by mixing 1 mM GDP, 1 mM $AlCl_3$ and 10 mM NaF with the protein). These proteins were subsequently applied to SEC and the fractions of the dimers were collected. The dimers contained (**A**) homodimers of unlabelled tagged MxA^{MO2}-AS, (**B**) heterodimers of unlabelled tagged MxA^{MO2}-AS and labelled untagged MxA^{MO2}-AS, and (**C**) homodimer of labelled untagged MxA^{MO2}-AS. Finally, these dimers were immobilized on the surface of the quartz slide for imaging experiments as described in **Method**. During the FRET measurement, whereas **A** emitted no FRET signals and **C** flew away and cannot be detected, only **B** was measured as expected.

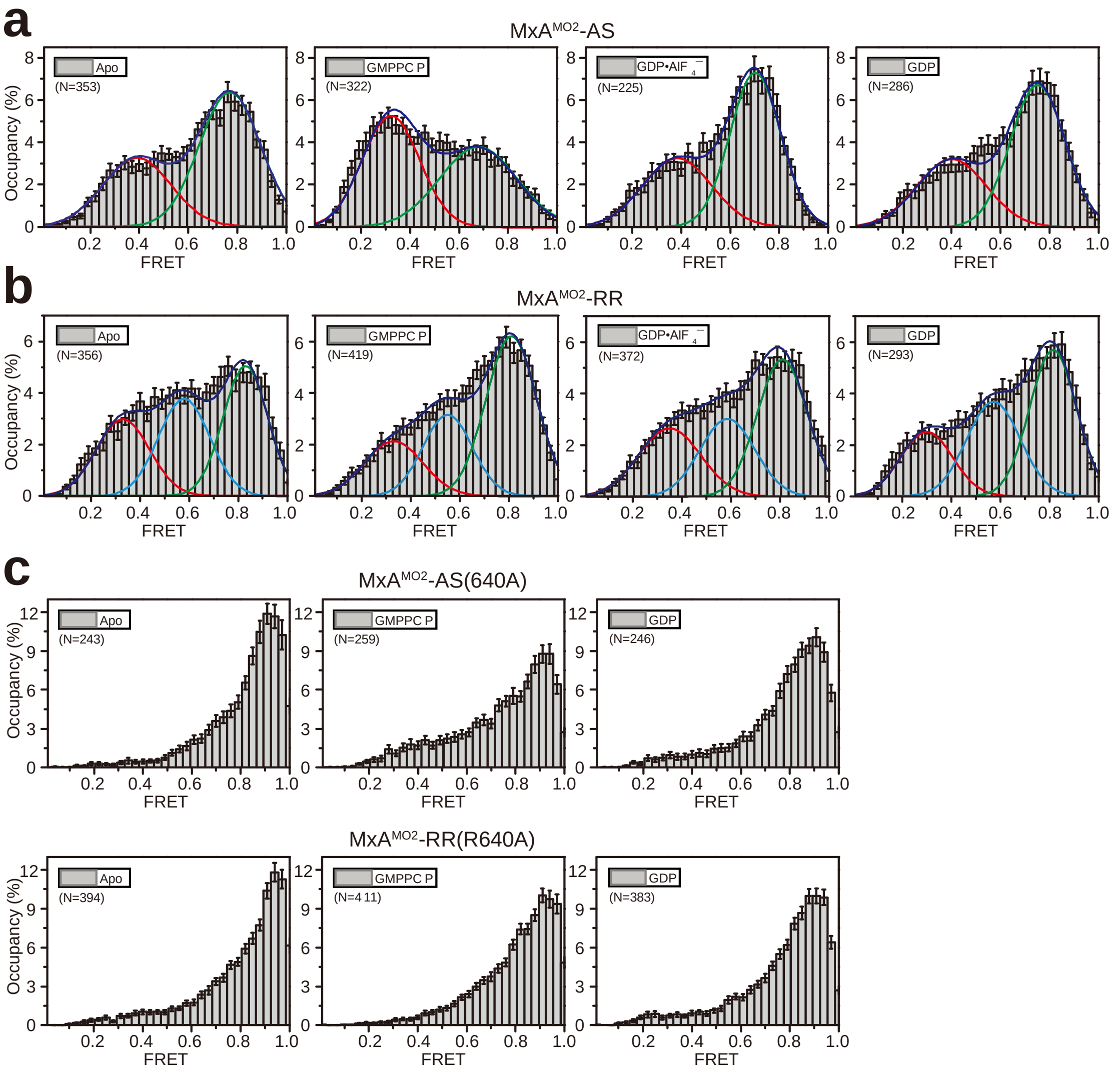

Supplementary Figure 8: Conformational dynamics of MxA molecules.

a, Histograms of FRET data from the mutant MxA^{MO2}-AS in different nucleotide-loading conditions. Decomposition of the FRET data resulted in two Gaussian curves representing distributions of a low-FRET state (red) and a high-FRET state (green). This is the repeated experiment result for Fig. 3**a**.

b, Histograms of FRET data from the mutant MxA^{MO2}-RR. Gaussian curves for low-, medium- and high-FRET states are coloured red, marine and green, respectively. This is the repeated experiment result for Fig. 4**a**.

c, Histograms of FRET data of MxA^{MO2}-AS(640A) (upper) and MxA^{MO2}-RR(R640A) (lower) in different nucleotide-loading conditions. This is the repeated experiment result for Fig. 5**a**. Error bars indicate standard deviation of 1,000 bootstrap samples of the FRET traces.

Supplementary Table 1: Data collection and refinement statistics.

	Monomeric MxA
**Data collection**	
Space group	P1 21 1
Cell dimensions	
a, *b*, *c* (Å)	104.18, 60.16, 153.92
α, β, γ (°)	90, 98.62, 90
Resolution (Å)	39.2-2.9 (3.0-2.9)*
R_{sym} or R_{merge}	0.122 (0.486)*
$I / \sigma I$	11.2 (3.3)*
Completeness (%)	98.84 (93.32)*
Redundancy	3.52 (4.9)*
**Refinement**	
Resolution (Å)	39.2-2.9
No. reflections	42599
R_{work} / R_{free}	0.2038 / 0.2634
No. atoms	
Protein	8449
Ligand/ion	0
Water	3
B-factors	
Protein	86.5
Ligand/ion	0
Water	51.2
R.m.s. deviations	
Bond lengths (Å)	0.010
Bond angles (°)	1.360

*Values in parentheses are for the highest resolution shell.

Supplementary Table 2: Gaussian component of MxA^{MO2}-RR in different conditions.

Gaussian Area	low-FRET state (%)	medium-FRET state (%)	high-FRET state (%)
Apo	26.3	35.5	38.2
GMPPCP	21.0	26.3	52.7
GDP•AlF_4^-	23.7	26.8	49.5
GDP	26.4	37.3	36.3

The area of each Gaussian composition in different nucleotide-loading conditions.

Supplementary Table 3: Dwell time of MxA^{MO2}-RR in different conditions.

Dwell time	low-FRET state (s)	medium-FRET state (s)	high-FRET state (s)
Apo	0.31±0.01	0.29±0.01	0.24±0.01
GMPPCP	0.25±0.02	0.31±0.01	0.35±0.03
GDP•AlF_4^-	0.28±0.04	0.34±0.03	0.57±0.11
GDP	0.30±0.03	0.25±0.01	0.31±0.02

Average dwell times in each FRET state in different nucleotide-loading conditions. The values are expressed as mean ± standard deviation.

Supplementary Reference

1 Thompson, J. D., Higgins, D. G. & Gibson, T. J. CLUSTAL W: improving the sensitivity of progressive multiple sequence alignment through sequence weighting, position-specific gap penalties and weight matrix choice. *Nucleic acids research* **22**, 4673-4680 (1994).
